# The Role of Educational Interactive Virtual Simulation App in Aesthetic Medicine and Cosmetic Dermatology Preclinical Skills

**DOI:** 10.1111/jocd.70496

**Published:** 2025-10-09

**Authors:** Haidar Hassan, Hassan Khalil, Bashar Shatta, Anna Maria Fenech Magrin, Ines Novo Pereira, Atif Matin

**Affiliations:** ^1^ Academic Plastic Surgery, Blizard Institute, Faculty of Medicine and Dentistry Queen Mary University of London London UK; ^2^ Faculty of Dental Medicine University of Porto Porto Portugal; ^3^ Egas Moniz Center for Interdisciplinary Research Egas Moniz School of Health & Science Almada Portugal

**Keywords:** aesthetic medicine, cosmetic dermatology, interactive virtual simulation, simulation‐based learning

## Abstract

**Background:**

The rapid progress in the field of aesthetic medicine and cosmetic dermatology drives the demand for skilled healthcare practitioners able to provide safe, complex treatments accurately and confidently. Traditional training approaches, which emphasize didactic teaching and little hands‐on experience, typically fail to prepare postgraduate students for these challenges. We hypothesized that interactive visual simulation may improve preclinical competency in aesthetic medicine and cosmetic dermatology.

**Aims:**

The aim of this study is to explore the impact of interactive visual simulations 3D app on the development of preclinical skills in aesthetic medicine and cosmetic dermatology.

**Methods:**

The study was designed as a mixed‐methods approach, which combined quantitative analysis of simulation performance data with quantitative feedback from participants. The study is based on a purposive sample of 25 healthcare professionals enrolled in aesthetic medicine postgraduate training programs at the Queen Mary University of London (QMUL). Data were collected through post‐training assessment questionnaires and performance metrics during simulations.

**Results:**

The findings clearly emphasized an improvement in the proficiency and confidence of participants who trained using interactive visual simulations. Trainees reported a greater sense of realism and immersion in their training, which helped them better understand the spatial relationships and anatomical structures involved in aesthetic procedures. Additionally, participants expressed increased confidence in their ability to perform these procedures on real patients.

**Conclusion:**

The study found that interactive visual simulation may help to improve aesthetic medicine and cosmetic dermatology preclinical competency. This technology has the potential to offer a better training experience than traditional approaches, resulting in more confident and skilled healthcare practitioners who can conduct difficult and safe aesthetic interventions.

## 1 Introduction

The field of aesthetic medicine and cosmetic dermatology has seen exponential growth over the last few decades, driven by technological advancements and increasing societal acceptance of cosmetic procedures [1]. Aesthetic medicine and cosmetic dermatology encompass a wide range of non‐surgical procedures designed to enhance the physical appearance of individuals, addressing issues such as skin aging, body contouring, and facial aesthetics [2, 3]. These procedures, which include botulinum toxin injections, dermal fillers, energy‐based treatments, topical growth factors, autologous plasma therapy, and chemical peels, require a high level of precision and expertise to ensure patient safety and satisfaction [3]. In today's world, it is undeniable that education and technology are inextricably linked. One powerful example is the use of a simulation environment, a longstanding approach that has gained prominence in educational settings with the advancement of technology, revolutionizing the way we learn [4, 5]. Simulation‐based education thrives in physical or technical settings, where it enhances student learning and assistant training. Its effectiveness has led to widespread adaptation across various professions, providing a hands‐on immersive learning experience [6, 7].

Despite the advancements in aesthetic medicine and cosmetic dermatology, traditional training methods for health practitioners remain largely unchanged. These methods typically involve theoretical coursework, observation, and limited hands‐on practice under supervision. However, this approach has several limitations. Theoretical knowledge alone cannot fully prepare health practitioners for the complexities of aesthetic and cosmetic procedures, which require a nuanced understanding of anatomy, precise hand‐eye coordination, and the ability to make quick, informed decisions during treatments. Additionally, the opportunity for hands‐on practice is often limited by the availability of suitable cases due to the need to ensure patient safety, resulting in insufficient practical experience for many trainees [8].

The potential benefits of interactive visual simulation in medical training have been demonstrated in various fields, including surgery, dentistry, and nursing. Studies have shown that simulation‐based training can enhance procedural skills, improve knowledge retention, and increase trainee confidence [9, 10, 11].

In the healthcare industry, simulation is utilized across three key domains [12]. First, it enables professionals to practice and assess technical skills using various approaches, from basic bench models to advanced virtual reality devices. Second, simulated patients have long been used to teach clinical skills and serve as a foundation for performance‐based evaluations. Lastly, simulation technologies facilitate team training, enhancing performance in high‐pressure complex scenarios [13, 14, 15].

Given the precision and unique challenges of aesthetic medicine and cosmetic dermatology, the application of simulation techniques in this field remains underexplored [9, 16]. Our study addresses this knowledge gap by investigating the effectiveness of interactive visual simulation in enhancing preclinical proficiency, paving the way for improved training and outcomes.

In addition, we sought to design simulation modules that could replicate common aesthetic procedures, using 3D modeling in comparison to 2D format. Ultimately, we aimed to integrate these modules into the training curriculum of the trainees and to measure their proficiency, accuracy, and confidence levels before and after using the simulation modules. In this direction, our research may contribute to laying the foundations for the development of guidelines and best practices for incorporating interactive visual simulation into aesthetic medicine and cosmetic dermatology educational programs, thereby improving student learning opportunities.

## 2 Material and Methods

### 2.1 Participants

Participants were recruited from Queen Mary University of London's (QMUL) aesthetic medicine postgraduate training programs (Years 1 and 2).

We included healthcare professionals enrolled in an aesthetic medicine postgraduate training Year 2023 to 2024 at the QMUL, willing to participate in the study and who were able to provide the informed consent. Exclusion criteria included: previous extensive experience with interactive visual simulations or refusal to consent. Participants were informed that participation was completely voluntary, and they could terminate their involvement at any time without any consequences.

A non‐probability sampling method was followed; that is, all the eligible subjects from the QMUL were invited to participate. Although we wanted our sample to be as representative as possible, data collection also needs to be feasible, and the eligibility criteria led to the final sample size of 25 trainees. The questionnaire was sent to the cohort of students of MSc Aesthetic Medicine QMUL Year 1 and 2. The recruited students were contacted electronically via e‐mail invitations.

### 2.2 Research Design and Setting

This study employed a participatory and interactive approach, following a simplified “action research cycle” that encompassed planning, observing, and reflecting [17]. This approach ensured the development of a mobile software program application (App), and its continuous improvement and adjustment based on the trainees' feedback. This study was conducted between February 2023 and July 2024. The mobile phone App development commenced in February 2023 and was completed in April 2023. Following planning, the data collection started in June 2023 and was completed in July 2024. Based on a positive perspective, this study used quantitative research methods to assess both the simulation performance data and the perspectives of the participants with regards to the novel teaching tool. The questionnaire was developed using insights from existing studies [6, 18, 19, 20, 21, 22], drawing on the PIETA Rubric [23], to ensure relevance and academic rigor. These studies shaped the thematic focus and item wording, aligning the instrument with current pedagogical and injection evaluation practices [4, 18, 19, 24, 25, 26]. A questionnaire consisting of a total of 12 questions was employed. The data set was analyzed using IBM SPSS Statistics, version 26 (IBM Corp., Armonk, NY, USA). For each question, descriptive statistics were employed to summarize the data: categorical variables were reported as frequencies and percentages. Bar charts and pie graphs were used to visually present the distribution of responses. Given the primarily descriptive nature of the analysis, no inferential tests or significance thresholds were applied. Results were presented using graphs.

#### 2.2.1 Planning

**FIGURE 1:**
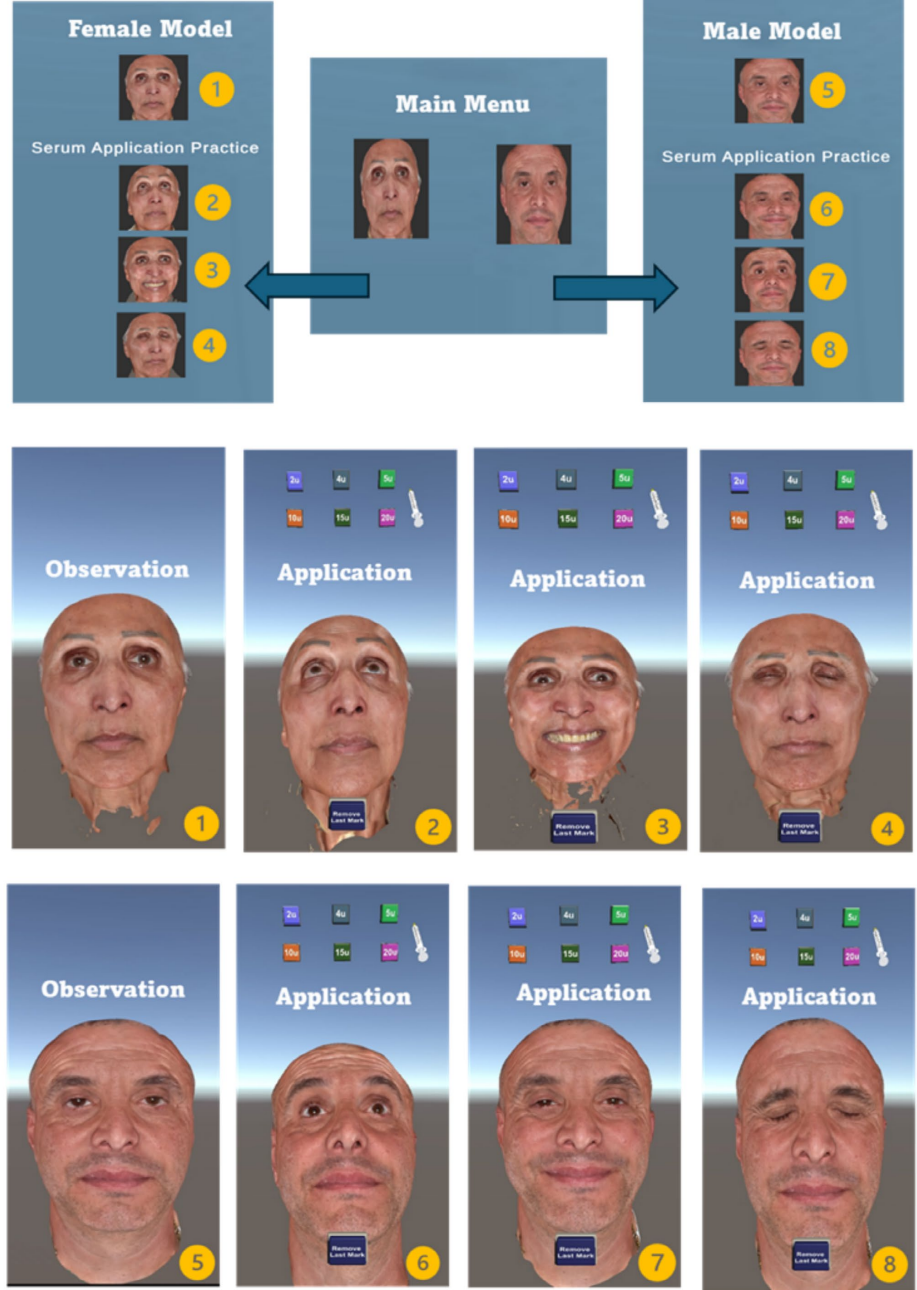
3D Simulation App user interface flowchart. Treatment planning and botulinum toxin injection pattern. 1, Female model observation—3D assessment of the whole face at rest. 2, Female model facial dynamic assessment of musculature (in animation—raising eyebrows) and treatment application targeting horizontal lines/frontalis muscle. 3, Female model facial dynamic assessment of musculature (in animation—smiling) and treatment application targeting lateral canthus/crow's feet. 4, Female model facial dynamic assessment of musculature (in animation—frowning) and treatment application targeting glabella. 5, Male model observation—3D assessment of the whole face at rest. 6, Male model facial dynamic assessment of musculature (in animation—raising eyebrows) and treatment application targeting horizontal lines/frontalis muscle. 7, Male model facial dynamic assessment of musculature (in animation—smiling) and treatment application targeting lateral canthus/crow's feet. 8, Male model facial dynamic assessment of musculature (in animation—frowning) and treatment application targeting glabella.

Initially, a cross‐platform application was developed, compatible with iOS and Android smartphones (iOS: https://apps.apple.com/us/app/3dam/id6472669610, Android: https://login.microsoftonline.com/569df091‐b013‐40e3‐86ee‐bd9cb9e25814/login). The objective was to get insights from the trainees of aesthetic medicine and cosmetic dermatology, with a specific focus on botulinum toxin procedures. The App incorporates interactive visual simulation in 3D. The links of the 3D app and 2D format were randomly distributed to the first‐ and second‐year aesthetic medicine students via email. The links invited the students to download the app on their iOS or Android devices. The software provides users with a secure means of practicing without incurring any avoidable dangers. It allows users to include botulinum toxin planning and injection patterns into 3D facial models, replicating real clinical scenarios (Figure 1).

#### 2.2.2 Observation

Following the postgraduate student's exposure to interactive 3D simulation and 2D module methodologies, they were instructed to fill out a questionnaire (Table 1). This was a self‐developed, closed‐ended 10‐point Likert scale. The scale consists of 12 questions, answered as follows:

**TABLE 1:** Questions from the survey categorized into their respective domains.

Domains	Questions
3D Model Visualization and Assessment	(Q1) How do you evaluate the visual similarity of the patient face on the 3D app model compared to the paper‐based 2D face model of the patient? (Q2) Do you think that the 3D training can better assist you in providing facial aging assessments in comparison to the 2D images? (Q3) Do you think 3D simulation app is useful to understand 3D structure of facial aging?
Clinical Skill Enhancement	(Q4) After training on a 3D model, has your ability to analyze skin conditions improved? (Q5) After training on a 3D model, has your injection treatment planning improved? (Q6) Do you believe that the 3D training has improved your skills to inject real patient? (Q7) Do you believe you have achieved a better understanding of injection procedures after training on 3D simulation app?
Procedural Efficiency and Planning	(Q8) Do you believe that the employment of a 3D simulation app is useful for reducing the operative time of a real procedure? (Q9) Do you think that the employment of 3D simulation models is useful for planning the real injection intervention?
Professional Development and Satisfaction	(Q10) Do you think that the 3D training has improved your confidence as an aesthetic and cosmetic health practitioner? Regardless of if you are an experienced practitioner or just starting a career (Q11) Do you believe your motivation to learn injection techniques has increased after using 3D simulation app? (Q12) How likely are you to recommend this 3D simulation app to a colleague?

0–1 = Strongly disagree, 2–4 = Disagree, 5 = Neutral, 6–8 = Agree, 9–10 = Strongly agree.

These items belong to four subscales that the participants were requested to evaluate, namely “3D Model Visualization and Assessment”, “Clinical Skill Enhancement”, “Procedural Efficiency and Planning”, and “Professional Development and Satisfaction”. A quantitative study was conducted by collecting responses.

#### 2.2.3 Reflection

An assessment of the App's effectiveness in enhancing postgraduate students' confidence and proficiency in performing aesthetic and cosmetic treatment was conducted using the collected data. The app's strengths and areas for improvement were identified based on participants' feedback. The iterative nature of the app development allowed for continuous improvement in meeting the educational requirements of aesthetic trainees, resulting in subsequent versions that were more effective in addressing their demands. Our objective was to create an adaptive learning resource that would enable adjustments to users' requirements and input in real‐world scenarios, utilizing the “action research cycle” [27].

#### 2.2.4 Ethical, Legal, and Regulatory Aspects

The study did not collect sensitive data (e.g., data revealing racial or ethnic origin, sexual orientation, and religious beliefs). Patients participated on a voluntary basis and signed the consent before the beginning of the study. There was no financial incentive for participants. The findings of this study were treated anonymously. The study group was fully committed to respecting the highest ethical and legal standards.

## 3 Results

A final total sample of 25 participants was recruited for the study. Of the participants who took part, 4 males (16%) and 21 females (84%) completed the study. The descriptive data analysis of the quantitative feedback offers insight into the effectiveness of the Virtual Simulation App from the perspective of postgraduate students. The percentages were calculated for each of the statements rated on a scale of 10 points. The data from the questionnaires were divided into subcategories with 0 to 1 falling under “Strongly Disagree”, 2 to 4 falling under “Disagree”, 5 being neutral, 6 to 8 falling under “Agree”, 9 to 10 falling under “Strongly Agree”.

### 3.1 Subscales

#### 3.1.1 3D Model Visualization and Assessment

**FIGURE 2:**
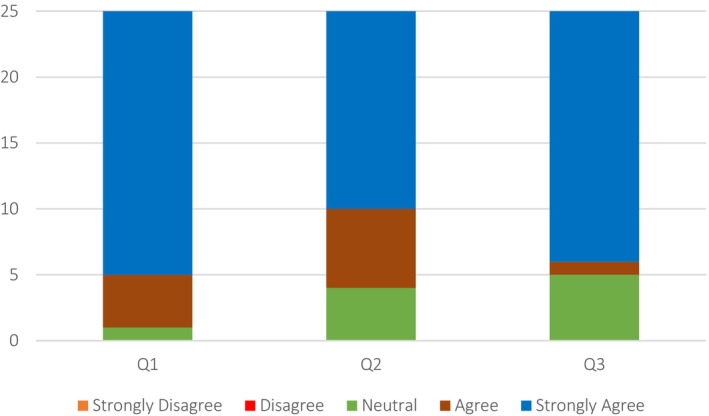
Quantitative Data results from questions (Q1–Q3) assessing 3D Model Visualization and Assessment efficacy. (Q1) How do you evaluate the visual similarity of the patient face on the 3D app model compared to the paper‐based 2D face model of the patient? (Q2) Do you think that the 3D training can better assist you in providing facial aging assessments in comparison to the 2D images? (Q3) Do you think 3D simulation app is useful to understand 3D structure of facial aging?

96% of the respondents either strongly agreed or agreed that the visual similarity between the 3D application model of the patient's face and that of the paper‐based 2D model was in fact similar (Figure 2, Q1). The remaining 4% remained neutral, neither agreeing nor disagreeing with the notion. 76% of the respondents strongly agreed that the 3D model improved their facial aging assessments compared to 2D face models, while another 20% agreed with the notion (Figure 2, Q2). The remaining 4% remained neutral to the idea. There was complete unanimity (100% of respondents) that the 3D simulation app proved useful in understanding the 3D structure of facial aging (Figure 2, Q3).

#### 3.1.2 Clinical Skill Enhancement

**FIGURE 3:**
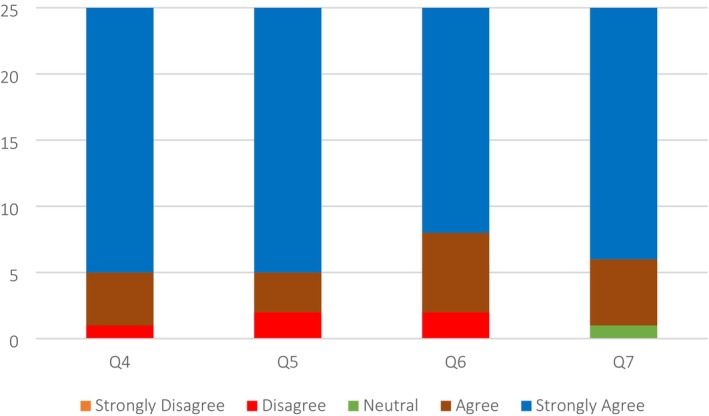
Quantitative Data results from questions (Q4–Q7) assessing Clinical Skill Enhancement. (Q4) After training on a 3D model, has your ability to analyze skin conditions improved? (Q5) After training on a 3D model, has your injection treatment planning improved? (Q6) Do you believe that the 3D training has improved your skills to inject real patient? (Q7) Do you believe you have achieved a better understanding of injection procedures after training on 3D simulation app?

The data shows that 84% of respondents either agreed or strongly agreed that the app enhanced the ability to analyze skin conditions (Figure 3, Q4), while 16% of respondents were neutral, neither agreeing nor disagreeing. This indicates an enhancement in the students' ability to analyze skin conditions following the delivery of training on a 3D Model. While 20% of respondents remained neutral to the question, 80% of respondents either strongly agreed (76%) or agreed (4%) that the 3D model improved their injection treatment planning, which indicates a positive sentiment to the proposition (Figure 3, Q5). 96% of respondents agreed, with the vast majority (80%) strongly agreeing with the assertion that the 3D training positively improved their skills to inject real patients. However, 4% disagreed with this statement (Figure 3, Q6). 76% of respondents strongly believed that the 3D simulation app helped them to achieve a better understanding of injection procedures, with another 12% agreeing, albeit 12% of the respondents disagreed (Figure 3, Q7).

#### 3.1.3 Procedural Efficiency and Planning

**FIGURE 4:**
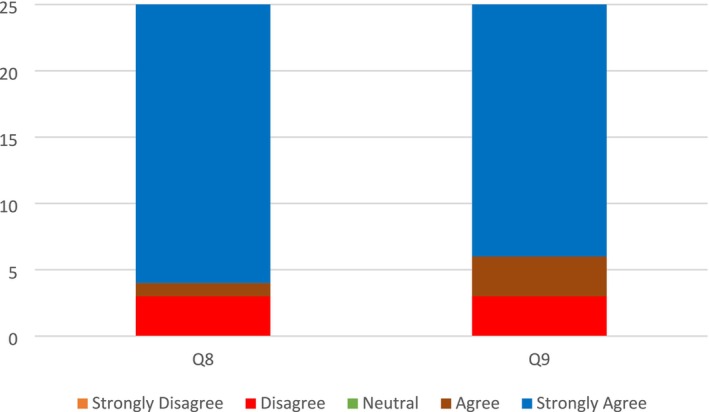
Quantitative Data results from questions (Q8, Q9) assessing Procedural Efficiency and Planning. (Q8) Do you believe that the employment of a 3D simulation app is useful for reducing the operative time of a real procedure? (Q9) Do you think that the employment of 3D simulation models is useful for planning the real injection intervention?

Although there was a slight decrease in those who felt strongly about this statement, 92% of the respondents agreed that the 3D app would be useful in reducing operative time during a real procedure, while 8% disagreed (Figure 4, Q8). Nevertheless, the consensus was that the 3D application has the potential to reduce operative time in a real procedure. 12% of the respondents disagreed with the notion, whereas 84% strongly agreed that the 3D simulation model was useful for planning real injection intervention, with another 4% agreeing with the idea (Figure 4, Q9).

#### 3.1.4 Professional Development and Satisfaction

**FIGURE 5:**
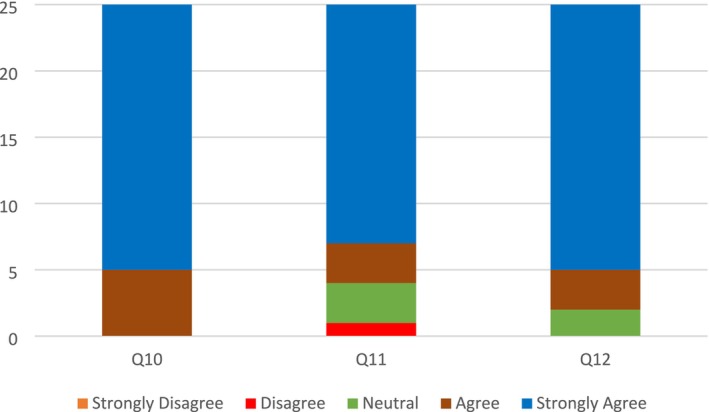
Quantitative Data results from questions (Q10–Q12) assessing Professional Development and Satisfaction. (Q10) Do you think that the 3D training has improved your confidence as an aesthetic and cosmetic health practitioner? Regardless of if you are an experienced practitioner or just starting a career. (Q11) Do you believe your motivation to learn injection techniques has increased after using 3D simulation app? (Q12) How likely are you to recommend this 3D simulation app to a colleague?

Despite 8% of respondents disagreeing with this notion, the majority strongly agreed (80%) or agreed (12%) that the 3D training model enhanced their confidence as aesthetic and cosmetic health practitioners, regardless of their experience in the field (Figure 5, Q10). 12% of the respondents felt neutral, with another 4% disagreeing that the 3D model increased their motivation to learn injection techniques. However, 84% of respondents strongly agreed (72%) or agreed (12%) that the 3D model led to an increase in their motivation to learn injection techniques (Figure 5, Q11). 92% of respondents positively indicated their likelihood to recommend the 3D app to a colleague, with only 8% still neutral, that is, neither unlikely nor likely to recommend the app to a colleague (Figure 5, Q12).

## 4 Discussion

Currently, the integration of simulation in different global contexts highlights its potential in training the next generation of healthcare professionals. The evidence provided by our study supports that digital tools have the potential to help bridge the theoretical‐practical gap in aesthetic medicine and cosmetic dermatology education. The findings demonstrated that a mobile app can effectively enhance clinicians' confidence and competence in performing aesthetic procedures, particularly botulinum toxin treatment. Our analysis is consistent with previous studies that demonstrated that medical students perceived realistic simulation as an effective teaching approach in different clinical settings.

The interactive features of the app, such as real‐time visualization of effects, provide an engaging and risk‐free environment for clinicians to experiment with various techniques and dosages [28]. This aspect is particularly crucial in aesthetic medicine, where precision and confidence are essential. The ability to visualize the outcomes of different procedures without the risk of patient harm allows health practitioners to refine their skills and approaches, leading to higher quality care [19].

The data indicating high levels of agreement among respondents across various aspects of the technology underscores its broad acceptance and perceived value among health practitioners. This consensus suggests that the 3D simulation technology is not only effective but also widely regarded as beneficial, which is important for its adoption and integration into standard practice. A core finding of the study is the effectiveness of 3D models in replicating real‐world conditions. This accuracy is vital for training purposes, as it ensures that the skills and techniques learned in the simulation can be directly applied to actual patient care [20]. The overwhelming agreement among participants about the accuracy of these models in representing facial features reinforces the app's utility in improving skills in skin analysis and treatment planning. Enhanced health practitioner confidence, as reported in the study, is another critical outcome [24]. Confidence in their abilities can lead health practitioners to perform procedures more effectively and with greater assurance, potentially improving patient outcomes [29]. Increased efficiency not only benefits the healthcare practitioners by allowing them to treat more patients, but also reduces the overall strain on medical facilities and resources [30, 31]. Moreover, the positive impact on motivation to learn injection techniques suggests that 3D simulation technology can be a powerful educational tool [6]. Engaged and motivated healthcare practitioners are likely to invest more effort in mastering new techniques, which can lead to continual improvement in practice standards and patient care.

One of the strengths of our study is the focus on more than one academic year, although it only covered one case scenario involving botulinum toxin injections. Research in aesthetic medicine and cosmetic dermatology is difficult as most respondents are self‐paying aesthetic and cosmetic healthcare practitioners who do not want to be involved in such assessments. Second, simulation can emulate numerous elements of clinical practice; it falls short of fully encompassing the intricacies and unpredictability inherent in real patient care. This limitation underscores the significance of utilizing simulation as an enhancement to, rather than a substitute for, conventional clinical training.

## 5 Conclusion

The integration of 3D simulation technology into aesthetic medicine and cosmetic dermatology practices is strongly supported by the evidence presented in this study. Its ability to enhance skills, boost confidence, and improve efficiency makes it a valuable asset for aesthetic and cosmetic health practitioners. While further research is necessary to fully explore the technology's potential, the current findings provide a solid foundation for its continued development and implementation. This study underscores the transformative potential of digital tools in aesthetic and cosmetic education and practice, paving the way for more innovative and effective approaches to clinician training and patient care.

## Author Contributions

All authors contributed to the study conception and design. Acquisition of data, analysis, and interpretation of data were performed by (H.H. and H.K.). The first draft of the manuscript was written by (H.H. and H.K.), and all authors commented on previous versions of the manuscript and provided critical revisions. All authors read and approved the final manuscript.

## Ethics Statement

The authors confirm that the ethical policies of the journal, as noted on the journal's author guidelines page, have been adhered to. All study procedures were conducted in accordance with the latest version of the Helsinki Declaration. Written consent was obtained from all participants after providing full explanations about the study procedures, benefits, and potential risks. The appropriate ethical review committee approval has been received from the Queen Mary Ethics of Research Committee (QMERC23.073).

## Conflicts of Interest

The authors declare no conflicts of interest.

## Data Availability

The data that support the findings of this study are available from the corresponding author upon reasonable request.
